# Spatiotemporal expression of the serine protease inhibitor, SERPINE2, in the mouse placenta and uterus during the estrous cycle, pregnancy, and lactation

**DOI:** 10.1186/1477-7827-8-127

**Published:** 2010-10-27

**Authors:** Schu-Rern Chern, Sheng-Hsiang Li, Chung-Hao Lu, Edmund I Tsuen Chen

**Affiliations:** 1 Department of Biotechnology and Laboratory Science in Medicine, National Yang-Ming University, Taipei, Taiwan; 2 Department of Medical Research, Mackay Memorial Hospital, Taipei, Taiwan; 3 Graduate Institute of Biotechnology, National Taipei University of Technology, Taipei, Taiwan

## Abstract

**Background:**

SERPINE2, also known as glia-derived nexin or protease nexin-1, belongs to the serine protease inhibitor (SERPIN) superfamily. It is one of the potent serpins that modulates the activity of the plasminogen activator (PA) and was implicated in tissue remodeling. In this study, we investigated the expression patterns of SERPINE2 in the mouse placenta and uterus during the estrous cycle, pregnancy, and lactation.

**Methods:**

SERPINE2 was purified from mouse seminal vesicle secretion using liquid chromatography (LC) and identified by LC/tandem mass spectrometry. The antiserum against the SERPINE2 protein was raised in rabbits. To reveal the uterine and placental expression of SERPINE2, tissues at various stages were collected for real-time PCR quantification, Western blotting, and immunohistochemical staining.

**Results:**

Serpine2 mRNA was the major PA inhibitor in the placenta and uterus during the estrous cycle, pregnancy, and lactation, although Serpine1 mRNA had higher expression levels than Serpine2 mRNA in the placenta. Plat seemed to be the major PA in the mouse uterus and placenta. Antiserum against the SERPINE2 protein specifically recognized two forms of SERPINE2 and an extra 75-kDa protein, which was probably a complex of SERPINE2 with a certain protease, from among thousands of protein components in the tissue extract as demonstrated by Western blotting. In the uterus, SERPINE2 was primarily localized in luminal and glandular epithelial cells but it also was detected in circular and longitudinal smooth muscle cells during the estrous cycle and lactation. It was prominently expressed in decidual stroma cells, the metrial gland, and endometrial epithelium of the pregnant uterus. In the placenta, SERPINE2 was expressed in trophoblasts of the labyrinth and spongiotrophoblasts. However, its expression was remarkably reduced in giant cells which existed in the giant cell-decidual junction zone. In contrast, prominent expression of SERPINE2 seemed to be detected on clusters of glycogen cells near the junction zone. In addition, yolk sac membranes also showed high expression of SERPINE2.

**Conclusions:**

These findings indicate that SERPINE2 is a major PA inhibitor in the placenta and uterus during the estrous cycle, pregnancy, and lactation. It may participate in the PA-modulated tissue remodeling process in the mouse placenta and uterus.

## Background

The mammalian uterus undergoes drastic tissue remodeling during the estrous cycle, implantation, and pregnancy. Tissue remodeling requires a fine-tuned balance between levels of proteases and their cognate inhibitors. The plasminogen activator (PA) system refers to the PA and its cognate inhibitors [1]. The PA is involved in tissue remodeling by converting abundant extracellular plasminogen into plasmin, an active protease, which degrades the extracellular matrix. The classical substrate of plasmin is fibrin, in fact, most other matrix proteins can be cleaved by this enzyme [1]. So far, two forms of PA, tissue-type (PLAT) and urokinase-type (PLAU) are reported. The activity of PA is modulated by several protease inhibitors that belong to the serine protease inhibitor (SERPIN) superfamily, such as SERPINA5, SERPINB2, SERPINE1, and SERPINE2 [2].

The PA system is associated with many physiological processes, including ovulation, embryogenesis, and embryo implantation in female reproductive tissues [1,3], and pathological processes, such as neoplasia [1]. How SERPIN modulates the proteolytic activities of PLAT/PLAU in reproductive tissue remodeling is of great importance.

The expression and activity of PLAT and PLAU were detected in female reproductive tissues, including the endometrium during cycling [4,5], implantation [6], and placentation [7-10]. Also, PLAU was found to be expressed during mouse placental development [10].

SERPINE1 was demonstrated to be present in human and mouse uteri during implantation [6,11], indicating that the PA inhibitor is involved in implantation. It was also detected in the placenta [8,9]. However, few studies have examined uterine SERPINE2 expression. SERPINE2, also known as glia-derived nexin or protease nexin-1, has broad anti-protease activity specific to serine proteases, including trypsin, thrombin, plasmin, prostasin [12], and PLAU [13]. It is widely expressed in various tissues [14]. Lin et al. reported that expression levels of SERPINE2 in the monkey endometrium and placenta during early pregnancy were weak or below the level of detection [15]. On the contrary, SERPINE2 was highly expressed in the human placenta throughout pregnancy [16]. In rats, *Serpine2 *mRNA was exclusively detected in endometrial stromal cells of the uterus, in particular on day 6.5 postcoitally, thus suggesting that it may be involved in the implantation process [17]. It seems that different species have different expression patterns for the *Serpine2 *gene. So far, no comprehensive study has exactly determined the expression of SERPINE2 in the murine placenta and uterus during the estrous cycle, pregnancy, and lactation. We therefore conducted this investigation to reveal the placental and uterine expression of the *Serpine2 *gene and the cellular localization of SERPINE2 protein in mice.

## Methods

### Animals and tissue collection

Specific pathogen-free outbred ICR mice were bred and maintained in the Animal Center of the Department of Medical Research, Mackay Memorial Hospital. Animals were treated according to institutional guidelines for the care and use of experimental animals. They were housed under controlled lighting (14 h of light, 10 h of dark) at 21~22°C and were provided with water and NIH-31 laboratory chow ad libitum. Normal 12-week-old adult male mice were used to purify the SERPINE2 protein as previously described [18]. The estrous cycle was staged by examining vaginal smears as described by Rugh [19]. The vaginal impedance was monitored by the MK-10A impedance checker (Muromachi Kikai, Tokyo, Japan) to work as a reference prior to doing vagina smear. The electric resistance of vagina has been used for determination of rat estrous cycle [20]. It also was useful for mouse to monitor the distinct phase of estrous cycle. The day when the vaginal plug was observed was designated day 0.5 of pregnancy. To investigate *Serpine2 *mRNA and protein expression in the placenta and uterus at various physiological stages, adult non-pregnant and pregnant female mice were sacrificed and their tissues removed. For each mouse, part of the tissue was stored in liquid nitrogen for mRNA and protein analyses, and another part was fixed in 10% (v/v) formaldehyde for the immunohistochemical analysis.

### RNA isolation, reverse transcription, and real-time polymerase chain reaction (PCR)

Total RNA was extracted from tissue homogenates treated with DNase I using an RNeasy Mini kit (Qiagen, Valencia, CA, USA). Five micrograms of total RNA was then reverse-transcribed into a 20-μl first-strand complementary (c)DNA pool using a High-Capacity cDNA Archive Kit (Applied Biosystems, Foster City, CA, USA) according to the manufacturer's instructions.

To examine *Serpin *gene expression, the real-time PCR was conducted. PCR primers (Table 1) were designed to cross the junction between the exon and intron. We used the hypoxanthine guanine phosphoribosyltransferase (*Hprt*) gene as the internal loading control to normalize the relative gene expression levels.

**Table 1:** Summary of real-time PCR primers

**Gene**^**a**^	Primer	Sequence	Position	Product size (bp)
*Serpina5*				
	F^b^	5'-TCTCCATTGAGGCTACCTACAAACT-3'	1071-1095	
	R^c^	5'-GTGCACCATCTCAGACAACTTGA-3'	1201-1179	131
*Serpinb2*				
	F	5'-TTCCGTGTGAACTCGCATGA-3'	664-683	
	R	5'-GGAAGCAACAGGAGCATGCT-3'	806-787	143
*Serpine1*				
	F	5'-CAGAGCAACAAGTTCAACTACACTGA-3'	810-835	
	R	5'-CAGCGATGAACATGCTGAGG-3'	915-896	106
*Serpine2*				
	F	5'-CAGATCATCAAGTCACGGCCT-3'	269-289	
	R	5'-ACCGTGGAGAGCTGCTTCTTT-3'	387-367	119
*Plat*				
	F	5'-AAGAGAGCAGCTCTGTTGGCAC-3'	1366-1387	
	R	5'-AATGGAGACGATGCCTCATGC-3'	1477-1457	112
*Plau*				
	F	5'-GAAGCGACCCTGGTGCTATG-3'	445-464	
	R	5'-TTTGCTAAGAGAGCAGTCATGCA-3'	526-504	82
*Hprt*				
	F	5'-GAATCACGTTTGTGTCATTAGTGAAA-3'	752-777	
	R	5'-TGCGCTCATCTTAGGCTTTGTA-3'	813-792	62

^a ^GenBank accession nos.: *Serpina5*, NM_172953; *Serpinb2*, NM_011111; *Serpine1*, NM_008871; *Serpine2*, NM_009255; *Plat*, NM_008872; *Plau*, NM_008873; *Hprt*, NM_013556

^b ^F, forward primer

^c ^R, reverse primer

Samples (*n *= 4 or 5) at each stage were separately analyzed. The PCR amplification efficiency for each gene was tested to ensure that it was equivalent to that of *Hprt mRNA *examined in a cDNA dilution series. The PCR was performed in a total volume of 20 μl, containing 50 ng of tissue cDNA, 150 nM each of the forward and reverse primers, and 10 μl of 2× SYBR Green Master Mix (Applied Biosystems). All reactions were performed in triplicate and run on an ABI/PRISM 7500 Fast Sequence Detector System (Applied Biosystems) under the following conditions: 50°C for 2 min, 95°C for 10 min, and then 40 cycles at 95°C for 15 s and 60°C for 1 min. The threshold cycle (Ct) was defined as the fractional cycle number at which the reporter fluorescence, i.e., the number of amplified copies, reached a fixed threshold. A melting curve analysis was run to verify that only a single product had formed in the reaction. The identity of the PCR products was confirmed by DNA sequencing. Relative quantification of mRNA expression was calculated by the 2^-ΔΔCt ^method [21].

### Protein purification and identification

To obtain the pure antigen to produce a highly specific and sensitive antiserum, the native SERPINE2 protein was purified from the mouse seminal vesicle secretion (SVS), since the mouse seminal vesicle prominently expresses SERPINE2 [14]. Adult male mice (10~12 weeks old) were sacrificed by cervical dislocation. The SVS was collected, centrifuged, and fractionated successively by ion-exchange chromatography on a diethylaminoethyl Sephacel (GE Healthcare Life Sciences, Piscataway, NJ, USA) column and gel-filtered on a Sephadex G-75 (GE Healthcare Life Sciences) column as previously described [18]. Peak 2 eluted from the Sephadex G-75 column was further subjected to ion-exchange high-performance liquid chromatography (HPLC) on a Protein PAK SP 5PW (Waters, Milford, MA, USA) column (7.5 cm × 7.5 mm). The column was eluted with a linear gradient of 0%~60% (w/v) 1.0 M NaCl in 20 mM sodium acetate at pH 6.0 at a flow rate of 1.0 ml/min for 50 min. The chromatogram is shown in Additional file 1, Figure S1. The protein concentration was determined using a bicinchoninic acid protein assay kit (Pierce, Rockford, IL, USA). The purified protein was identified by sodium dodecylsulfate polyacrylamide gel electrophoresis (SDS-PAGE) on a 15% gel slab (8.2 × 7.3 × 0.075 cm). Protein bands on the SDS-PAGE gel were excised and subjected to in-gel digestion with trypsin following our previously described method [22]. The result is shown in Additional file 1, Figure S1.

### Production of the specific antiserum

Antisera against SERPINE2 were produced in New Zealand white rabbits. The purified protein in normal saline (0.4 mg/ml) was emulsified with Freund's complete adjuvant (1:1, v/v) (Sigma-Aldrich, St. Louis, MO, USA). In total, 2 ml of the mixture was injected subcutaneously in multiple sites in a rabbit. Two rabbits were boosted twice every 3 weeks with the mixture of the same amount of purified protein and Freund's incomplete adjuvant (1:1, v/v) (Sigma-Aldrich). Antiserum was collected 10 days after the last injection.

Two hundred micrograms of the purified SERPINE2 protein was conjugated to AminoLink beads according to the manufacturer's instructions (Pierce). Antisera against SERPINE2 were adsorbed by the conjugated beads to remove the specific antibody against SERPINE2. The treated antiserum was used as the control antiserum.

### Western blotting

Tissue extract proteins were resolved using SDS-PAGE on a 10% gel slab (8.2 × 7.3 × 0.075 cm) and were transferred to a nitrocellulose membrane for immunostaining according to a previously described method [23]. Membranes were blocked with 10% (w/v) skim milk in phosphate-buffered saline (PBS) (blocking solution) for 2 h, and then incubated with anti-SERPINE2 antiserum (1: 5000) or monoclonal anti-α-tubulin (1: 15,000, Sigma-Aldrich) in blocking solution for 1 h at room temperature. After gentle agitation in four changes of PBS for 15 min each, they were immunoreacted with horseradish peroxidase (HRP)-conjugated goat anti-rabbit immunoglobulin G (IgG) (1: 10,000, GE Healthcare Life Sciences) or HRP-conjugated anti-mouse IgG (1: 15,000; Cell Signaling Technology, Beverly, MA, USA) in blocking solution for 1 h. Immunoreactive bands were revealed using an enhanced chemiluminescent (ECL) substrate according to the manufacturer's instructions (Pierce).

### Immunohistochemical staining

Tissues were collected, fixed in formalin, embedded in paraffin, and cut into 5-μm sections. After the slides were deparaffinized and hydrated, they were placed in a plastic slide holder filled with the antigen retrieval AR-10 solution (BioGenex, San Ramon, CA, USA), soaked in a 70°C water bath, rapidly boiled to > 95°C, and maintained for 15 min. While cooling to room temperature for 30 min, the slides were treated with 3% (v/v) H_2_O_2 _in PBS for 15 min, blocked with 10% (v/v) normal goat serum in PBS (blocking solution) for 1 h at room temperature, and then incubated with anti-SERPINE2 antiserum or antiserum pretreated with SERPINE2-conjugated beads diluted 1: 700 in the blocking solution at 4°C for 16 h. After washing, the slides were treated with biotin-conjugated goat anti-rabbit IgG (~3 μg/ml) (Zymed Laboratories, South San Francisco, CA, USA) in blocking solution for 1 h at room temperature. The slides were washed again and then incubated with HRP-conjugated streptavidin (~1 μg/ml) (Zymed Laboratories) in blocking solution for 40 min at room temperature. Protein signals were detected by 3-amino-9-ethylcarbazole staining (Zymed Laboratories). The slides were then counterstained with hematoxylin (Vector Laboratories, Burlingame, CA, USA) and photographed using a Zeiss AxioImager Z1 microscope system equipped with CCD camera and an automated acquisition system (TissueGnostics, Vienna, Austria).

### Statistical analysis

Data are presented as the mean ± standard deviation (SD). Differences were analyzed by the one-way analysis of variance (ANOVA) followed by Bonferroni *post hoc *test using InStat software (GraphPad, San Diego, CA, USA). A *p *value of < 0.05 was considered significant.

## Results

### Expression of the SERPINE2 protein in the mouse uterus during the estrous cycle

**Figure 1:**
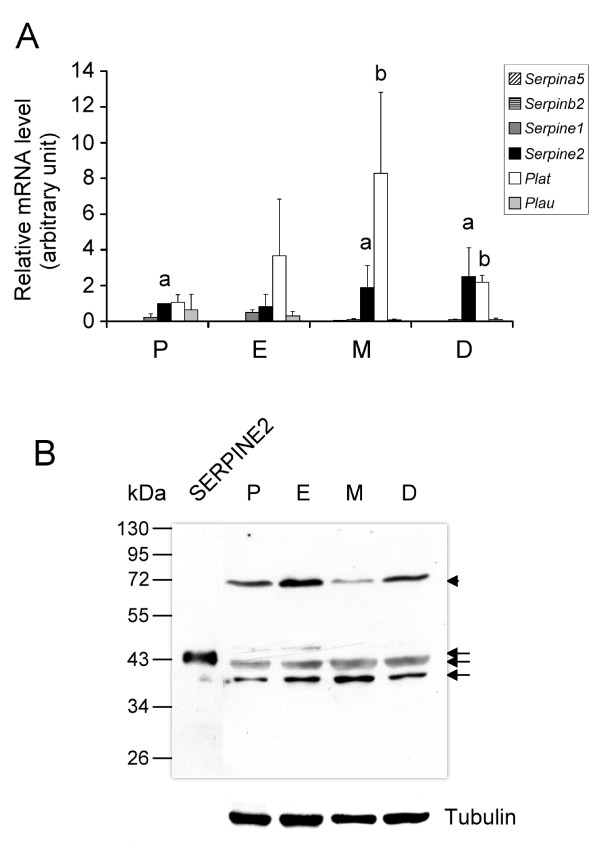
**Expression of the plasminogen activator (PA) and PA inhibitors in the mouse uterus during the estrus cycle**. (A) Real-time PCR was conducted to evaluate gene expression levels. Relative levels of mRNA were normalized to *Hprt mRNA *levels. Data are presented as the mean of four individual experiments, and error bars represent the standard deviation (SD). ^a ^Significant difference compared to other *Serpin *mRNA levels (*p *< 0.05). ^b ^Significant difference compared to *Plau *mRNA levels (*p *< 0.001). (B) Expression of the SERPINE2 protein in the cycling uterus. Forty-five micrograms of total protein prepared from the homogenates of uterine tissue at proestrus (P, lane 2), estrus (E, lane 3), metestrus (M, lane 4), and diestrus stages (D, lane 5) was analyzed by Western blotting. The purified SERPINE2 protein (30 ng) was loaded as the positive control (lane 1). The arrowhead indicates a possible protein complex of SERPINE2 and a certain protease. Arrows indicate the isoform proteins of SERPINE2. The α-tubulin protein level is shown as the loading control.

We analyzed the gene expression of PAs and their cognate inhibitors, i.e., *Serpina5*, *Serpinb2*, *Serpine1*, and *Serpine2*, in the mouse uterus during the estrous cycle by real-time PCR. As shown in Figure 1A, *Plat *mRNA was apparently the most highly expressed PA in the mouse uterus. *Serpinea5 *and *Serpinb2 *had very minor signals or were not detected. However, *Serpine2 *mRNA was the most prominently expressed PA inhibitor, while *Serpine1 *mRNA was next. *Serpine2 *mRNA's expression was significant among PA inhibitors examined in the cycling uterus, being especially prominent in metestrus and diestrus uteri.

To produce specific antibodies, we purified the SERPINE2 protein from the SVS (see "Methods" and Additional file 1 for details) as the antigen. As revealed by Western blotting, the antiserum against SERPINE2 recognized the purified 43-kDa band. Two forms, about 40- and 42-kDa bands, of SERPINE2 were detected from thousands of protein components in the uterine tissue extract collected from different phase of estrous cycle (Figure 1B, arrows). An approximately 75-kDa band was also seen at the four stages of the cycling uterus (Figure 1B, arrowhead). The 75-kDa band was probably the complex of SERPINE2 and its cognate protease, which were demonstrated in previous studies [12,14,24,25]. Thus, two forms of SERPINE2 and an extra 75-kDa SERPINE2-protease complex were expressed in the mouse uterus. No signal was seen (data not shown) when the antiserum was removed from the blots and reprobed with the antiserum that was pretreated with SERPINE2-conjugated beads (control antiserum), indicating the high specificity of the antibody.

**Figure 2:**
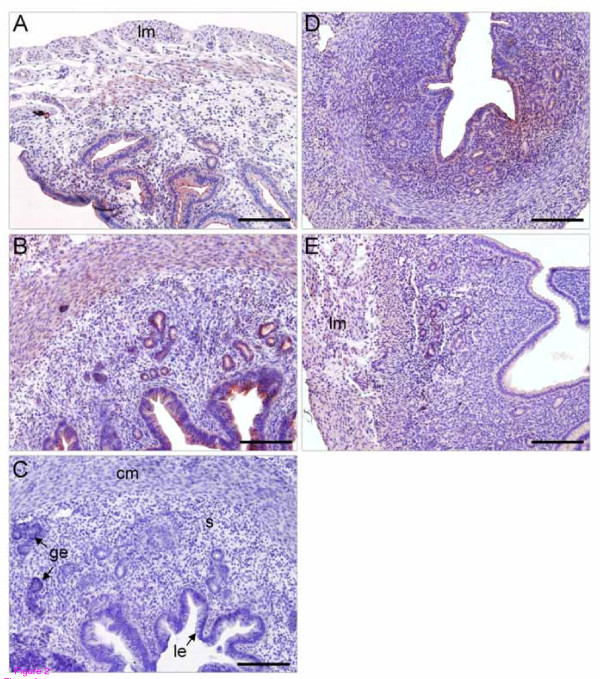
**Immunolocalization of SERPINE2 in the mouse uterus at different stages of the estrous cycle**. Uterine sections during proestrus (A), estrus (B, C), metestrus (D), and diestrus stages (E) were incubated with anti-SERPINE2 antiserum or the antiserum pretreated with SERPINE2 protein (control) (C), and then treated with biotin-conjugated goat-anti-rabbit IgG and horseradish peroxidase-conjugated streptavidin (red). For contrast, specimens were further stained with hematoxylin (blue). Photographs were taken under bright-field illumination. Bar = 200 μm. cm, circular muscle; ge, glandular epithelium; le, luminal epithelium; lm, longitudinal muscle; s, stroma.

To reveal the cellular localization of the SERPINE2 protein in the mouse uterus during the estrous cycle, an immunolocalization study was conducted using specific anti-SERPINE2 antiserum. The SERPINE2 protein was primarily immunolocalized to luminal and glandular epithelial cells and was weakly expressed in circular and longitudinal smooth muscle cells at proestrus, estrus and diestrus, but was nearly undetectable at metestrus. Signals from stromal cells were relatively weaker (Figure 2A,B,E) except at metestrus (Figure 2D). However, when slides were immunostained with control antiserum, no signal was detected (Figure 2C).

### Expression of the SERPINE2 protein in the mouse uterus and placenta during pregnancy

**Figure 3:**
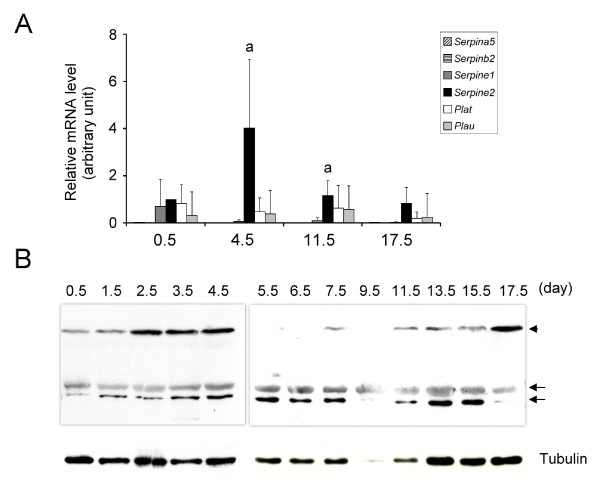
**Expression of *Serpine2 *mRNA and protein in the mouse uterus during pregnancy**. (A) A real-time PCR analysis was conducted to quantitate the *Serpine2 *gene's expression levels at various pregnancy stages. The relative levels of mRNA were normalized to *Hprt mRNA *levels. Data are presented as the mean ± standard deviation (SD) from four independent experiments. ^a ^Significant difference compared to other *Serpin *mRNA levels (*p *< 0.05). (B) SERPINE2 protein expression in the uterus was examined at various pregnancy stages. Forty-five micrograms of total protein prepared from various days of a pregnant uterus was analyzed by Western blotting. The arrowhead indicates a possible protein complex of SERPINE2 and a certain protease. Arrows indicate the isoform proteins of SERPINE2. The α-tubulin protein level is shown as the loading control.

To assess the gene expression of PAs and PA inhibitors in the pregnant uterus, a quantitative real-time PCR was performed using several stages of uterine tissues during pregnancy. As shown in Figure 3A, among the PA inhibitors, *Serpine2 *mRNA seemed to be most highly expressed in the pregnant uterus, while *Plat *and *Plau *mRNA showed no differences in expression levels among the different stages examined. The SERPINE2 protein was detected at various stages in the pregnant uterus by Western blotting. The 75-kDa protein was again detected in uterine tissues (Figure 3B). These findings confirm the expression of *Serpine2 *mRNA and protein in the gravid uterus.

**Figure 4:**
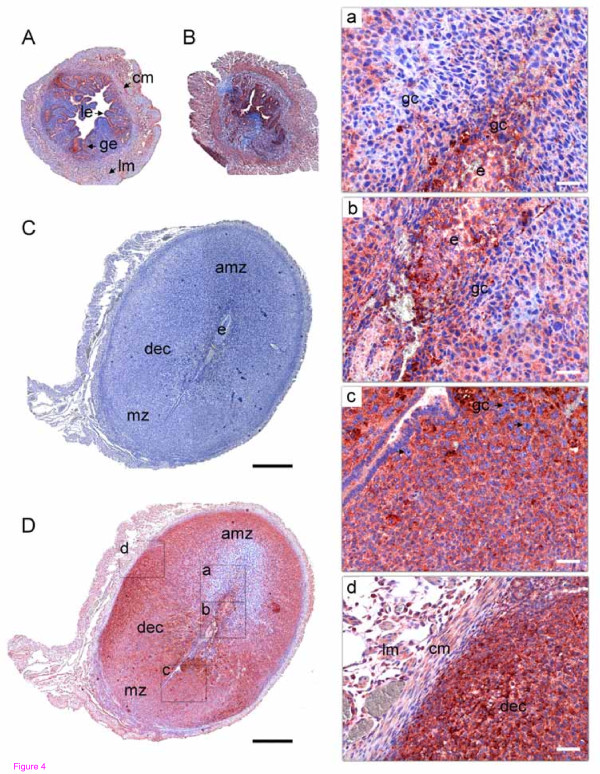
**Immunolocalization of SERPINE2 in the mouse uterus during early pregnancy**. Uterine sections prepared from a pregnant uterus on days 0.5 (A), 4.5 (B), and 7.5 (C, D, and a-d) were histochemically stained as described in Figure 2. A control section stained with control antiserum is shown (C). The black and white bars indicate 500 and 50 μm, respectively. amz, antimesometrial zone; cm, circular muscle; dec, decidua; e, embryo; gc, giant cells; ge, glandular epithelium; le, luminal epithelium; lm, longitudinal muscle; mz, mesometrial zone.

Uterine expression of SERPINE2 was detected early in the pregnancy of mice (Figure 4). On days 0.5 and 4.5, SERPINE2 was primarily expressed in luminal and glandular epithelia, while a visible protein signal was also detected in the circular and longitudinal layers of the myometrium (Figure 4AB). It should be noted that stronger protein expression was found during the implantation period on day 4.5 of pregnancy (Figure 4B). On day 5.5, SERPINE2 was prominently expressed in stromal cells during decidualization (data not shown). On day 7.5, trophoblast giant cells appeared among decidual stromal cells in the antimesometrial zone; however, their SERPINE2 protein expression was obviously weaker (Figure 4D). Trophoblast giant cells that stemmed from the embryo invaded decidual cells (Figure 4D, a, b). Giant cells that appeared in the antimesometrial zone showed significantly weaker SERPINE2 protein expression, while the situation was opposite in the mesometrial zone (Figure 4D, c). In contrast to the antimesometrial stroma, the mesometrial stroma strongly expressed SERPINE2. In addition to prominent SERPINE2 expression in decidual cells, visible signals were seen in the circular and longitudinal layers of the myometrium (Figure 4D, d).

**Figure 5:**
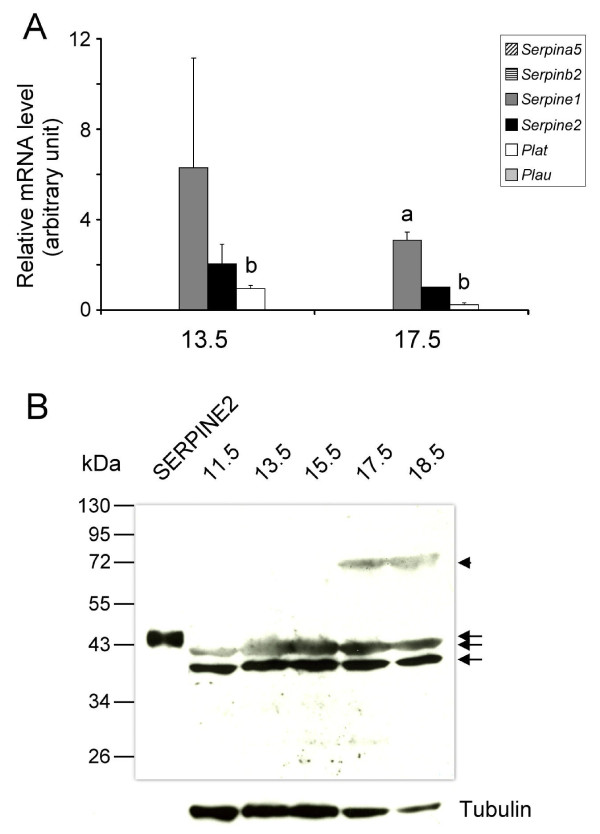
**Expression of *Serpine2 *mRNA and protein in the placenta**. (A) Placental expression of *Serpine2 *mRNA. Quantitative real-time PCR was conducted to analyze gene expression levels. The relative levels of mRNA were normalized to *Hprt mRNA *levels. Data are presented as the mean of three individual experiments, and error bars represent the standard deviation (SD). ^a ^Significant difference compared to *Serpine2 *mRNA levels (*p *< 0.001). ^b ^Significant difference compared to *Plau *mRNA levels (*p *< 0.001). (B) Placental SERPINE2 expression. Forty-five micrograms of total protein prepared from the homogenates of placental tissue was analyzed by Western blotting. The arrowhead indicates a possible protein complex of SERPINE2 and a certain protease. Arrows indicate the isoform proteins of SERPINE2. The α-tubulin protein level is shown as the loading control.

In the placenta, *Plat *mRNA seemed to be the most highly expressed PA as demonstrated by a quantitative real-time PCR (Figure 5A). *Serpinea5 *and *Serpinb2 *mRNAs were almost not detected, while *Serpine1 *mRNA was more highly expressed than was *Serpine2 *mRNA (Figure 5A). The SERPINE2 protein was also detected in the placenta by Western blotting (Figure 5B).

**Figure 6:**
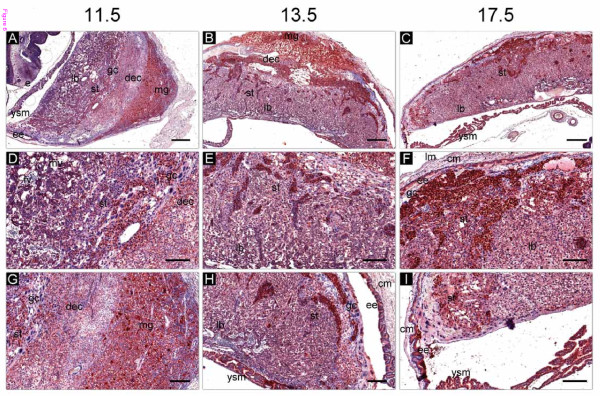
**Immunolocalization of SERPINE2 in the pregnant uterus and placenta on days 11.5, 13.5, and 17.5 of pregnancy**. Uterine sections were immunostained as described in Figure 2. Bars indicate 500 μm in A-C and 200 μm in D-I. cm, circular muscle; dec, decidua; e, embryo; ee, endometrial epithelium; fv, fetal vessel; gc, giant cells; gly, glycogen cells; lb, labyrinth; lm, longitudinal muscle; mg, metrial gland; mv, mother vessel; st, spongiotrophoblast; ysm, yolk sac membrane.

SERPINE2 was expressed in various cells in the placenta and uterus during mid- and late-pregnancy. In the uterus, it was prominently expressed in the decidua, metrial gland, and endometrial epithelium and relatively more weakly in the uterine myometrium on days 11.5, 13.5, and 17.5 of pregnancy (Figure 6). In the placenta, the protein was localized in trophoblasts of the labyrinth and spongiotrophoblasts. However, giant cells in the junction zone showed significantly lower protein expression (Figure 6). It was interesting to note that high levels of SERPINE2 were found in clusters of glycogen cells in the junction region (Figure 6D,E,F). SERPINE2 was also highly expressed on yolk sac membranes throughout the pregnancy (Figure 6 and data not shown).

### Expression of the SERPINE2 protein in the mouse uterus during lactation

**Figure 7:**
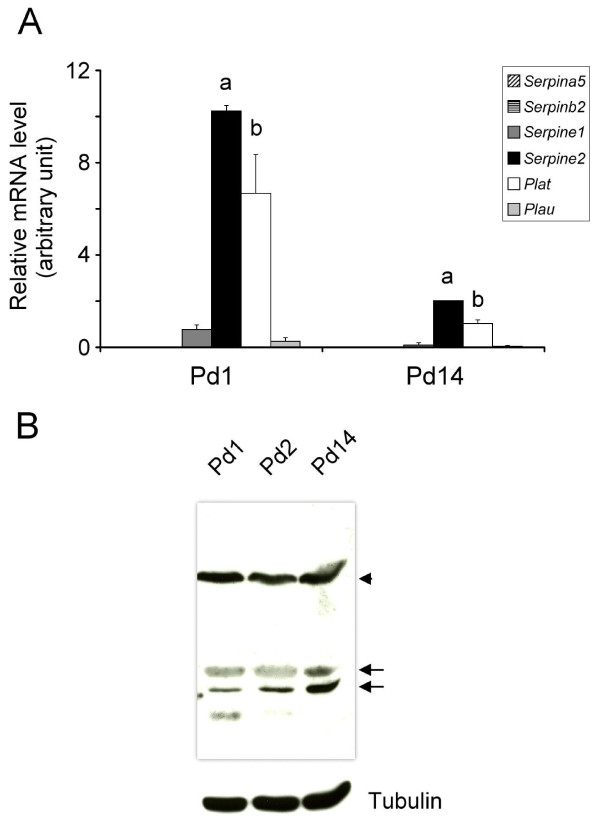
**Expression of *Serpine2 *mRNA and protein in the uterus during lactation**. (A) Expression of *Serpine2 *mRNA in the postnatal uterus. Quantitative real-time PCR was conducted to analyze gene expression levels. The relative levels of mRNA were normalized to *Hprt mRNA *levels. Data are presented as the mean of three individual experiments, and error bars represent the standard deviation (SD). ^a ^Significant difference compared to other *Serpin *mRNA levels (*p *< 0.001). ^b ^Significant difference compared to *Plau *mRNA levels (*p *< 0.001). (B) Uterine SERPINE2 expression after birth. Forty-five micrograms of total protein prepared from the homogenates of uterine tissue was analyzed by Western blotting. The arrowhead indicates a possible protein complex of SERPINE2 and a certain protease. Arrows indicate the isoform proteins of SERPINE2. The α-tubulin protein level is shown as the loading control. Pd1, postnatal day 1; Pd2, postnatal day 2; Pd14, postnatal day 14.

**Figure 8:**
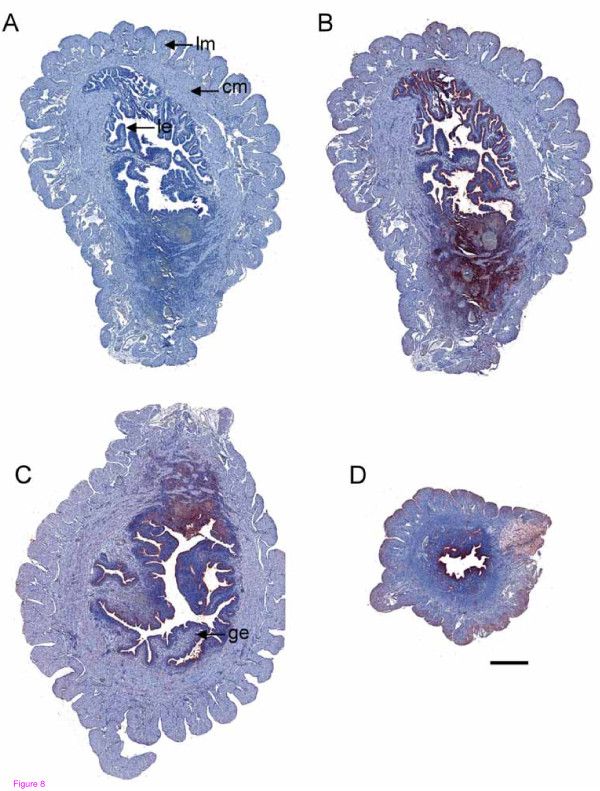
**Immunolocalization of SERPINE2 in the uterus during lactation**. Uterine sections were immunostained as described in Figure 2. The uterus on postnatal day 1 stained with control antiserum is shown (A). Uteri on postnatal days 1 (B), 2 (C), and 14 (D) were stained with anti-SERPINE2 antiserum. Bar = 500 μm. cm, circular muscle; ge, glandular epithelium; le, luminal epithelium; lm, longitudinal muscle.

*Serpine2 *and *Plat *mRNAs had a tendency to be highly expressed in the mouse uterus during lactation (Figure 7A). The SERPINE2 protein was consistently detected in the postnatal uterus by Western blotting (Figure 7B). Immunohistochemical staining showed that the SERPINE2 protein was strongly expressed in the luminal epithelium in the uterus at postnatal days 1 and 2 (Figure 8B,C). Endometrial SERPINE2 expression was also high even on postnatal day 14 (Figure 8D). The circular and longitudinal layers of the myometrium had weak SERPINE2 expression on postnatal days 1 and 2 in addition to the part around large vessels which had very high SERPINE2 expression levels (Figure 8B,C). However, SERPINE2 expression in the longitudinal muscle layer showed an increased signal on postnatal day 14 (Figure 8D).

## Discussion

In this study, we demonstrated that SERPINE2 is one of the major PA inhibitors that exists in the mouse placenta and uterus, where it is extensively expressed in various cell types. SERPINE2 is highly expressed in decidual stroma cells during embryo implantation and throughout placentation. It is expressed in labyrinthine trophoblasts and spongiotrophoblasts in the placenta. Intriguingly, giant cells expressed less SERPINE2 in the junction zone, while a group of glycogen cells immediately near the giants cells prominently expressed SERPINE2.

Giant cells in the mesometrial zone, which are differentiated from cells of the ectoplacental cone [26,27], largely expressed SERPINE2, while giant cells' SERPINE2 expression in the antimesometrial zone was low on day 7.5 of early pregnancy (Figure 4). Cells of the ectoplacental cone are precursors of the spongiotrophoblast layer. Indeed, SERPINE2 was prominently expressed in the spongiotrophoblast layer (Figure 6).

SERPINE2 can regulate the activity of serine proteases; thus, it is most likely involved in tissue remodeling. Tissue remodeling is an important biological event for many reproductive processes occurred in the ovary, uterus, and placenta, such as, follicle growth, ovulation, estrous cycle, implantation, and placentation. SERPINE2 is previously demonstrated to be upregulated primarily in dominant follicles during follicle growth [28,29] and during ovulation [30]. However, relatively little work has been done in the uterus. Although, in rats, *Serpine2 *mRNA is exclusively expressed in the endometrial stroma and is upregulated at the time of implantation [17], the *Serpine2 *mRNA and protein, in mice, is primarily detected in the glandular and luminal cells and is also upregulated at the implantation. The results suggest a role for SERPINE2 in regulating tissue remodeling during implantation.

The cell migration and invasion, the well-known events during placentation, is tightly regulated by proteases and their cognate protease inhibitors [27]. SERPINE2 is widely expressed in various cell types throughout placentation, indicating that this protein is involved in tissue remodeling during placental development.

Our SERPINE2 antiserum detected two major SERPINE2 isoforms in the uterus and placenta. In a protein database search conducted with Basic Local Alignment Search Tool (BLAST) algorithms (http://www.ncbi.nlm.nih.gov/BLAST) against a non-redundant database using the SERPINE2 protein sequence (Swiss-Prot Q07235) as the query, three isoforms were revealed with the accession numbers of gb|EDL16269.1|, gb|EDL16267.1|, and gb|EDL16268.1|. The theoretical molecular masses for the three isoforms were 30,812, 35,668, and 44,206 Da, respectively. They are not processed by a signal peptidase. Thus, the mature protein would have a smaller molecular mass. However, the three proteins recognized by the anti-SERPINE2 antiserum had larger molecular masses, indicating they may be the glycosylated forms. In fact, SERPINE2 was demonstrated to be expressed as two glycoprotein forms [31]. Many alternatively spliced gene products of SERPINE2 can also be seen on the NCBI AceView website (http://www.ncbi.nlm.nih.gov/IEB/Research/Acembly/). Previous reports also supported the existence of multiple forms of the SERPINE2 protein among mouse tissues [32].

Previous studies demonstrated that SERPINE2 can form a complex of approximately 75 kDa with PLAU [14,24,25]. Also, SERPINE2 was shown to act as a suicide inhibitor of prostasin through formation of an inactive SDS-stable complex with it [12]. Thus, the 75-kDa band detected by the anti-SERPINE2 antiserum may be the complex of SERPINE2 and a certain protease. Prostasin was detected in the monkey placenta [15]. Recently, prostasin was demonstrated to have the ability to regulate human placental trophoblast cell proliferation [33].

Although *Serpine2 *mRNA and the SERPINE2 protein are highly expressed in the mouse placenta, *Serpine1 *mRNA seems to be the major PA inhibitor found there. Similarly, *SERPINE1 *mRNA has higher expression levels than those of *SERPINE2 *mRNA in the human placenta throughout the three trimesters as demonstrated by quantitative real-time PCR (our unpublished data). The human SERPINE1 protein was intensely stained in trophoblasts invading the decidua and myometrium and was suggested to be a marker for invading trophoblasts [34], while the human SERPINE2 protein was primarily observed in the chorionic membrane and trophoblastic epithelium [16].

In our study, we found that *Serpinb2 *mRNA was not expressed or was below detection in the placenta and uterus during pregnancy. However, the SERPINB2 protein is expressed in the human placenta [34,35]. SERPINB2 was detected in the cytoplasm of villous syncytiotrophoblasts but not of villous cytotrophoblasts or invading trophoblasts [34].

Our anti-SERPINE2 antibody was very sensitive in detecting tissues in which there was trace expression of SERPINE2, even better than the commercial antibody which uses an *Escherichia coli*-expressed protein as the immunogen (data not shown). Weak or very low levels of SERPINE2 in the monkey endometrium and placenta in a previous study [15] may have resulted from the fact that they used the peptide or *E. coli*-expressed protein as the immunogen which is often not in the native conformation of a protein. We used a purified native protein as the immunogen; thus, we had a more-specific and -sensitive antiserum.

In an in situ hybridization study, rat *Serpine2 *mRNA was found to be primarily localized in endometrial stromal cells of the rat uterus [17]. We found that our anti-SERPINE2 antibody could cross-react well to rat uterine SERPINE2. Rat uterine SERPINE2 expression and localization were primarily in the endometrial epithelium, similar to the mouse uterus during the estrous cycle (Additional file 2, Figure S2). In fact, SERPINE2 was also detected in the human uterine endometrium, myometrium (our unpublished data), and placenta [16]. Thus, SERPINE2 must play some roles in uterine tissue remodeling in the PA system, since SERPINE2 is the major PA inhibitor in the uterus.

In conclusion, the SERPINE2 protein is the most highly expressed PA inhibitor in the mouse uterus but is second one in the placenta. The cellular localization of SERPINE2 in the mouse placenta and uterus suggests that SERPINE2 may play important roles in PA-modulated tissue remodeling.

## Competing interests

The authors declare that they have no competing interests.

## Authors' contributions

SRC carried out the Western blotting and real-time PCR analyses, and drafted the manuscript. SHL participated in the design of the study and helped draft the manuscript. CHL carried out protein purification, antibody production, and immunohistochemistry. EITC conceived of the study, and participated in the project design and coordination. All authors read and approved the final manuscript.

## Supplementary Material

Additional file 1 **Supplemental Figure 1**. Purification and identification of SERPINE2 from the mouse seminal vesicle fluid. Click here for file

Additional file 2 **Supplemental Figure 2**. Expression of the SERPINE2 protein in the rat uterus. Click here for file
