# Supplementary material for: Spatiotemporal expression of the serine protease inhibitor, SERPINE2, in the mouse placenta and uterus during the estrous cycle, pregnancy, and lactation
Source: Reprod Biol Endocrinol. 2010 Oct 27;8:127. doi: 10.1186/1477-7827-8-127 (PMC2987947; doi:10.1186/1477-7827-8-127)
Supplement: Additional file 1 — Supplemental Figure 1. Purification and identification of SERPINE2 from the mouse seminal vesicle fluid. [file 1477-7827-8-127-S1.PDF]

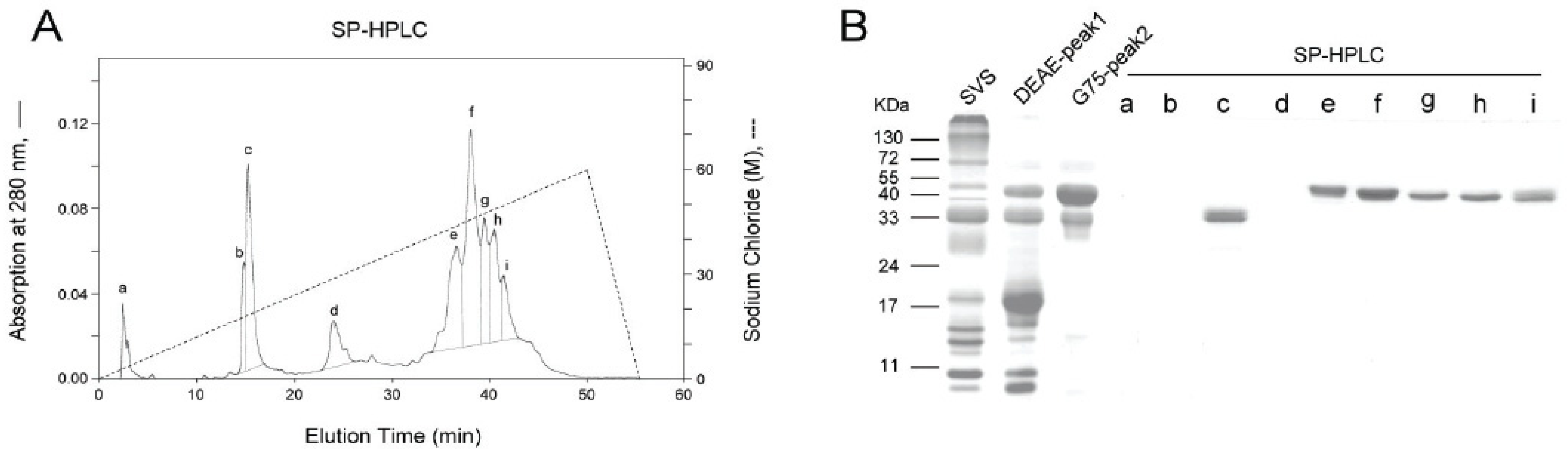

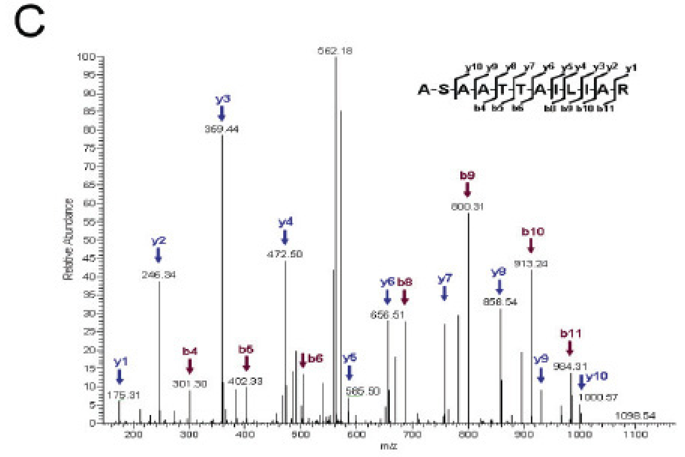

D

MNWHFPFFIL TTVTLYSVHS QFNSLSLEEL GSNTGIQVFN QIIKSRPHEN
VVVSPHGIAS ILGMLQLGAD GKTKKQLSTV MRYNVNGVGK VLKKINKAIV
SKKNKDIVTV ANAVFLRNGF KMEVPFAVRN KDVFQCEVQN VNFQDPASAS
ESINFWVKNE TRGMIDNLLS PNLIDGALTR LVLVNAVYFK GLWKSRFQPE
STKKRTFVAG DGKSYQVPML AQLSVFRSGS TRTPNGLWYN FIELPYHGES
ISMLIALPTE SSTPLSAIIP HITTKTIDSW MNTMVPKRMQ LVLPKFTAVA
QTDLKEPLKA LGITEMFEPS KANFTKITRS ESLHVSHILQ KAKIEVSEDG
TKASAATTAI LIARSSPPWF IVDRPFLFSI RHNPTGAILF LGQVNKP

Additional file 1 (pdf) Supplemental figure 1: Purification and identification of SERPINE2 from the mouse seminal vesicle fluid.

Nine peaks (a~i) obtained from liquid column chromatography of the fresh preparation of soluble seminal vesicle secretions (SVSs) were resolved by high performance liquid chromatography (HPLC) on a cation-exchange column at the final purification step (Fig. S1, A). Each of the representative samples at various steps of purification was resolved on a reducing SDS-PAGE gel (Fig. S1, B). Each lane on the gel is explained as follows: lane 1, 15 μg of SVS; lane 2, 15 μg of fraction I of DEAE-Sephacel chromatography; lane 3, 10 μg of peak 2 of G-75 gel filtration; lane 4~12, 3 μg of peaks a~i of HPLC. Peaks e to i yielded one broad ~43 kDa band. They were separately excised and digested in-gel with trypsin, and the resulting tryptic peptides were purified and subjected to liquid chromatography-mass spectrometry (LC/MS/MS) analysis. A representative MS/MS spectrum of a peptide from peak e is shown (Fig. S1, C). The amino acid sequence of the peptide is listed. The mass of each b- and y-ion is indicated. The results showed that each of the e-to-i peak samples had significant homology to the SERPINE2 protein with the tryptic peptides matching >40% of the putative protein sequences (Fig. S1, D). The cDNA-deduced amino acid sequence of mouse SERPINE2 is shown. The matched tryptic peptides in the LC/MS/MS analyses are underlined (Fig. S1, D).
