# Supplementary material for: Spatiotemporal expression of the serine protease inhibitor, SERPINE2, in the mouse placenta and uterus during the estrous cycle, pregnancy, and lactation
Source: Reprod Biol Endocrinol. 2010 Oct 27;8:127. doi: 10.1186/1477-7827-8-127 (PMC2987947; doi:10.1186/1477-7827-8-127)
Supplement: Additional file 2 — Supplemental Figure 2. Expression of the SERPINE2 protein in the rat uterus. [file 1477-7827-8-127-S2.PDF]

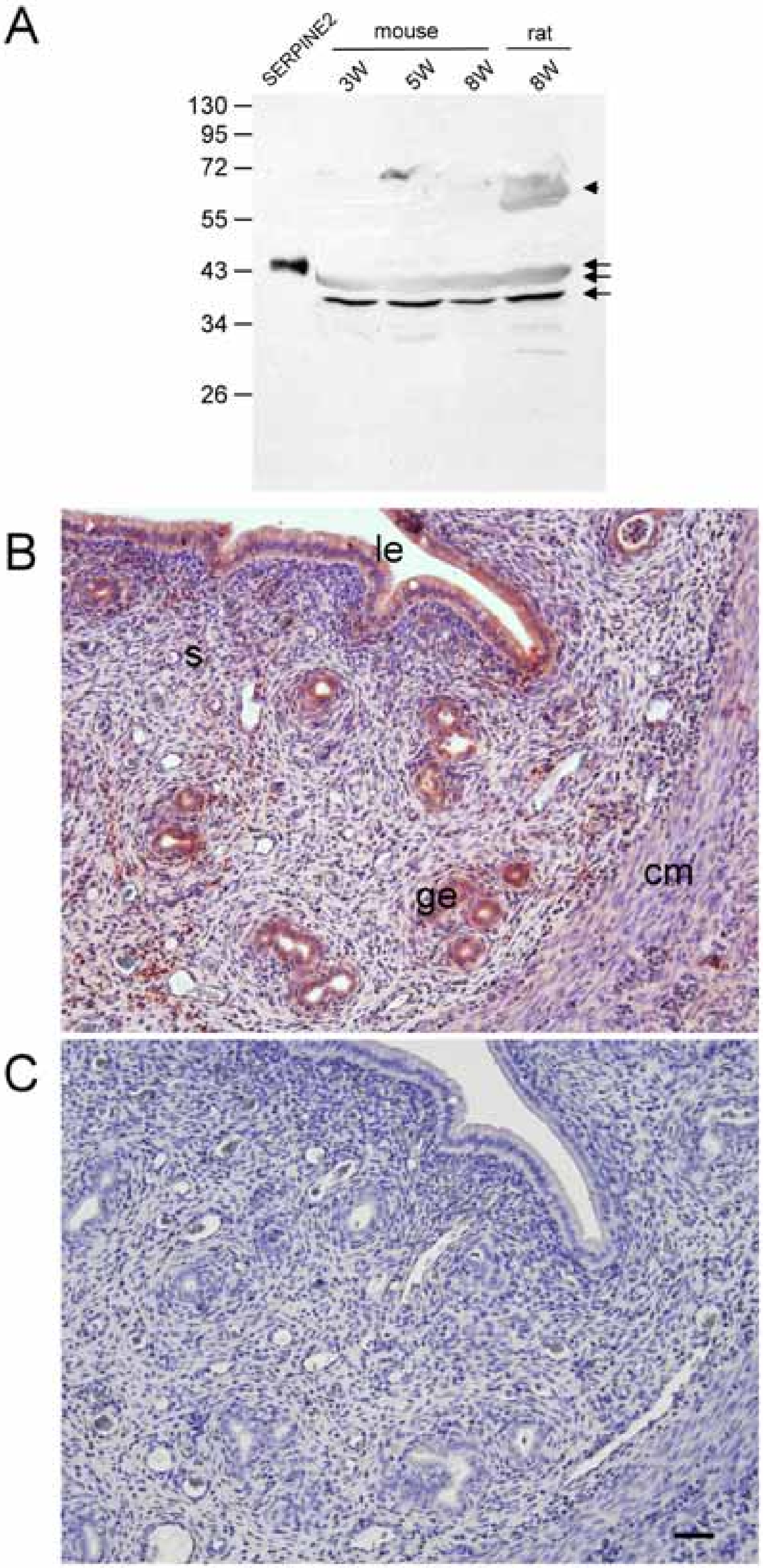

Additional file 2 (pdf) Supplemental figure 2: Expression of the SERPINE2 protein in the rat uterus.

The antiserum against mouse SERPINE2 could recognize the rat SERPINE2 with the similar blotting pattern to the mouse samples (Fig. 2S, A). Forty-five micrograms of total protein prepared from the homogenates of uterine tissue was analyzed by Western blotting. The purified mouse SERPINE2 protein (30 ng) was loaded as the positive control (lane 1). Lane 2, 3W, mouse uterus at 3 weeks old; lane 3, 5W, mouse uterus at 5 weeks old; lane 4, 8W, mouse uterus at 8 weeks old; lane 5, 8W, rat uterus at 8 weeks old. The arrowhead indicates a possible protein complex of SERPINE2 and a certain protease. Arrows indicate the isoform proteins of SERPINE2. The antiserum also was applied to stain the formalin-fixed and paraffin-embedded tissue slides prepared from rat uterus at the proestrus stage. The biotin-conjugated goat-anti-rabbit IgG and horseradish peroxidase-conjugated streptavidin were applied and the substrate 3-amino-9-ethylcarbazole (red) was used for signal development. The rat SERPINE2 protein was prominently immunolocalized to luminal and glandular epithelial cells and weakly expressed in stromal cells. In addition, visible signal was also detected in circular smooth muscle cells (Fig. 2S, B). However, when slides were immunostained with control antiserum, no signal was detected (Fig. 2S, C). For contrast, specimens were further stained with hematoxylin (blue). Photographs were taken under bright-field illumination. Bar = 50 μm. cm, circular muscle; ge, glandular epithelium; le, luminal epithelium; s, stroma.
